# A Multiplicity of Perspectives

**DOI:** 10.3201/eid2812.AC2812

**Published:** 2022-12

**Authors:** Byron Breedlove

**Affiliations:** Centers for Disease Control and Prevention, Atlanta, Georgia, USA

**Keywords:** art science connection, emerging infectious diseases, art and medicine, about the cover, public health, urbanization, cities, Rafael Barradas, Urban Landscape (Paisaje Urbano), a multiplicity of perspectives, urban populations, synanthropic wildlife, migrating birds, domestic and wild animals sold in markets, house pets, spillover, zoonotic infections, zoonoses, Uruguay

**Figure:**
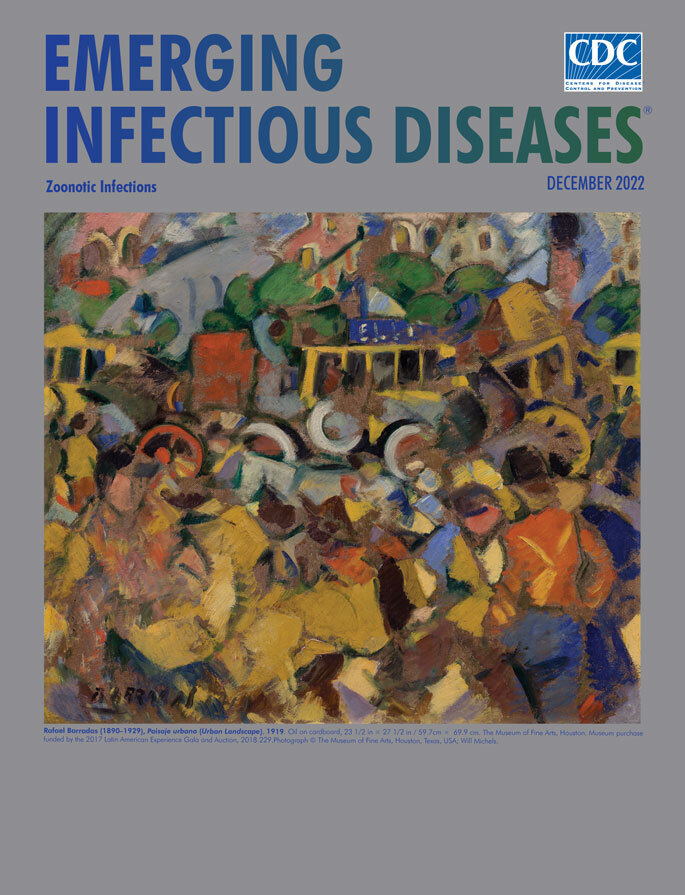
**Rafael Barradas (1890–1929), *Urban Landscape (Paisaje urbano)*, 1919.** Oil on cardboard, 23 1/2 in × 27 1/2 in / 59.7cm × 69.9 cm. The Museum of Fine Arts, Houston. Museum purchase funded by the 2017 Latin American Experience Gala and Auction, 2018.229. Photograph © The Museum of Fine Arts, Houston, Texas, USA; Will Michels.

Finding a point of reference within the jumble of images that comprise the frenzied street scene sprawled across* Urban Landscape (Paisaje urbano), *this month’s cover image*, *may initially prove challenging. A kaleidoscopic assortment of wedges, semicircles, rectangles, and other shapes seems to vibrate, morph, and blur. Swaths and streaks of color compete for attention. Pedestrians in the foreground jostle and nudge one another. To the left, a woman grasps a shopping basket. On the right, several uniformed soldiers pass among the crowd. Between them, indistinct images suggest a bustling scrum of people engaged in their daily routines. A stream of equestrians and yellow horse-drawn carriages, perhaps taxis, flows across the middle of the image. The few trees, green blots perched on a hill, and some patches of pale blue sky are the only nods to the natural world. 

It is not improbable that some viewers might also imagine the sounds and smells from this teeming thoroughfare or experience a tinge of claustrophobia from the crush of the crowd. If so, then its creator, Uruguayan artist Rafael Barradas, would have been pleased. 

Born in 1890 to Spanish parents in Montevideo, Uruguay, Barradas did not receive formal academic training or study art. His biography from the National Museum of Visual Arts, Uruguay, notes that Barradas learned his craft more informally, participating in gatherings with intellectuals and collaborating as an illustrator in newspapers and magazines published in Montevideo and Buenos Aires. 

In 1913, Barradas traveled to Europe and settled in Barcelona, Spain, where he met a number of artists and poets, including a kindred spirit, the painter Joaquín Torres-García, also from Uruguay. They “were among the preeminent Uruguayan artists to establish a vital connection with Europe’s prewar vanguards,” according to the Museum of Fine Arts, Houston, Texas, USA. The allure of futurism, cubism, and synchromism in art led Barradas and Torres-García “to propose ‘vibrationism’—essentially an art reflecting a unity of the senses—as a unique contribution to Modernist theory.”

Within the European avant-garde art community of that time, art historian M. Lluïsa Faxedas Brujats explains that vibration referred to the “physical phenomenology of light and to the assumed vibration of colours,” the comparison between light and sound that lead to “all kinds of research into synaesthesia,” and a connection “between the individual and his or her environment.” Gabriel Peluffo Linari, who researches Latin American art, describes vibrationism as having a “syncretic language with a wide range of colors” that “presented a multiplicity of perspectives” and “combined elements of cubism and Italian futurism.” 

During this somewhat short phase of his career (by 1924 Barradas had moved on from vibrationism and was mostly painting landscapes), the artist was striving to appeal to as many senses as possible in his colorful, complex, abstract paintings. The Museum of Fine Arts, Houston, uses musical terminology to describe the syncretic elements of this work, noting, “*Urban Landscape* reflects the clamorous city streets of Madrid, where Barradas had settled in 1919, with sound and motion distilled in a colorful fugue.”

When *Urban Landscape *was painted more than a century ago, according to United Nations’ data, only 20% of the world's population lived in cities; in less developed countries, that percentage was around 5%. In October 2022, the World Bank estimates that “some 56% of the world’s population―4.4 billion inhabitants—live in cities.” The United Nations recently projected that by 2050, 68% of people will live in urban areas. Urbanization and the accompanying loss of natural habitat for animals have amplified the spillover of zoonoses―infections that are naturally transmissible from animals to humans―since Barradas’ lifetime. As urban populations increase, so does human contact with synanthropic wildlife, migrating birds, domestic and wild animals sold in markets, and house pets, which in turn contributes to the spread of zoonotic infections. Plus, the overcrowding of people within megacities further propagates human-to-human spread of infections that have zoonotic origins but can now spread directly among people. 

Infectious disease specialist Carl-Johan Neiderud writes that “Urbanization leads to many challenges for global health and the epidemiology of infectious diseases. New megacities can be incubators for new epidemics, and zoonotic diseases can spread in a more rapid manner and become worldwide threats.” Integral to the global response to zoonotic infections is the concept of One Health, which CDC describes as “a collaborative, multisectoral, and transdisciplinary approach—working at the local, regional, national, and global levels—with the goal of achieving optimal health outcomes recognizing the interconnection between people, animals, plants, and their shared environment.” Such “a multiplicity of perspectives” from experts working in multiple disciplines and sectors can bolster public health preparedness and response for the next zoonotic pandemic.
